# Contribution of Red Wine Consumption to Human Health Protection

**DOI:** 10.3390/molecules23071684

**Published:** 2018-07-11

**Authors:** Lukas Snopek, Jiri Mlcek, Lenka Sochorova, Mojmir Baron, Irena Hlavacova, Tunde Jurikova, Rene Kizek, Eva Sedlackova, Jiri Sochor

**Affiliations:** 1 Department of Food Analysis and Chemistry, Faculty of Technology, Tomas Bata University in Zlín, Vavreckova 275, CZ-760 01 Zlín, Czech Republic; lsnopek@ft.utb.cz (Lu.S.); ihlavacova@ft.utb.cz (I.H.); evsedl@seznam.cz (E.S.); 2 Department of Viticulture and Enology, Faculty of Horticulture, Mendel University in Brno, Valtická 337, CZ-691 44 Lednice, Czech Republic; lenka.sochorova@mendelu.cz (Le.S.); mojmir.baron@mendelu.cz (M.B.); sochor.jirik@seznam.cz (J.S.); 3 Institute for Teacher Training, Faculty of Central European Studies, Constantine the Philosopher University in Nitra, Drazovska 4, SK-949 74 Nitra, Slovakia; tjurikova@ukf.sk; 4 Department of Human Pharmacology and Toxicology, Faculty of Pharmacy, University of Veterinary and Pharmaceutical Sciences Brno, Palackeho 1946/1, CZ-612 42 Brno, Czech Republic; kizekr@vfu.cz

**Keywords:** red wine, human, health, alcohol, phenolic compounds, antioxidants

## Abstract

Wine consumption has been popular worldwide for many centuries. Based on in vitro and in vivo studies, a certain amount of everyday wine consumption may prevent various chronic diseases. This is due, in part, to the presence and amount of important antioxidants in red wine, and, therefore, research has focused on them. Wine polyphenols, especially resveratrol, anthocyanins, and catechins, are the most effective wine antioxidants. Resveratrol is active in the prevention of cardiovascular diseases by neutralizing free oxygen radicals and reactive nitrogenous radicals; it penetrates the blood-brain barrier and, thus, protects the brain and nerve cells. It also reduces platelet aggregation and so counteracts the formation of blood clots or thrombi. The main aim of this review is to summarize the current findings about the positive influence of wine consumption on human organ function, chronic diseases, and the reduction of damage to the cardiovascular system.

## 1. Introduction

Red wine is popular worldwide and is beneficial due to the presence and amount of its compounds. The tradition of winemaking and wine consumption has been known for many centuries. The ancient Romans knew the health benefits of wine and popularized it [1]. The main product of grapes is wine. Wine is composed mainly of water, carbohydrates, organic acids, minerals, alcohol, polyphenols, and aromatics [2]. Wine contains substances that have a significant effect on cardiovascular diseases and on some chronic diseases [3].

Antioxidants are necessary for good cardiovascular function. They can be found in many plants, such as fruits (and their derivative products i.e., jams, juices, wine, etc.) and vegetables. Their presence in food and drink reduces the risk of cardiovascular diseases, some cancers, and diabetes [3,4]. Red wine consumption has been shown to decrease the blood pressure of hypertensive patients [5].

One of the most notable classes of wine compounds is polyphenols. The polyphenol composition and exact content are dependent on factors, such as the grape variety. White wines usually contain less polyphenols than red ones. The total polyphenol content in white wine is in terms of hundreds of mg GAE.L^−1^ (gallic acid equivalents) [6]. Red wine total polyphenol content is in terms of thousands of mg GAE.L^−1^. According to some studies [4], red wine polyphenols reduce the risk of cardiovascular diseases and have a positive impact on individual human organs.

The most important polyphenols in red wine are resveratrol, anthocyanins, catechins, and tannins (proanthocyanidins and ellagitannins) [7]. Resveratrol has a biological activity, plays an important role in cardiovascular diseases, and is present in a restricted number of foods, such as red wine and wine grapes. Its cardioprotective effects include improving endothelial function and glucose metabolism, reducing inflammation, and regulating blood lipids. Other wine polyphenols are an integral part of these actions and contribute positively to the beneficial effects of wine [5].

The aim of this overview is to summarize the various wine components, their antioxidant properties, significant polyphenols and their health benefits in relation to the cardiovascular system, and the effect on individual organs.

## 2. Red Wine and Its Antioxidants

The antioxidants found in many red purple berry fruits (grapes of red wine, Aronia, etc.) are categorized as individual polyphenols. The activity of berry fruit polyphenols in reducing the risk of cardiovascular diseases has been studied by Kim et al. [8]. The results showed the beneficial effects of polyphenols on heart function, reduction of cardiovascular diseases, arteriosclerosis and heart attacks, and reduced risk of hypertension and diabetes. Schini-Kerth et al. [9] examined the positive effect of regular consumption of food and drinks rich in polyphenols, such as red wine, tea, chocolate, and vegetables, on endothelial cells. Qureshi et al. [10] conducted a study about the foods and beverages contributing to antioxidant intake in a group of women aged 50 to 69 years. Women with a higher antioxidant intake (major contributors were coffee, tea, red wine, blueberries, walnuts, oranges, cinnamon, and broccoli) were at a lower risk for cardiovascular diseases, heart arrhythmia, hypertension, and diabetes. Women with regular red wine consumption were found to have the lowest risk.

One of the important and studied polyphenols of wine is resveratrol. Resveratrol, lipophilic 3,4′,5-trihydroxystilbene, found in grapes and red wines, exhibits a variety of pharmacological properties. The amount of resveratrol differs depending on the variety, geographical location, time of harvest, maturity, and health of the grapes [5].

Resveratrol, in some research, has shown cardioprotective benefits in humans, but Tome-Carneiro et al. [11] suggest that resveratrol’s positive effects are overestimated because of its low concentration in foods and low bioavailability in humans. These ideas are both supported and disproved by many clinical studies. In addition, long-term excessive intake of resveratrol decreases the activity of some isoenzymes, which may be damaging for human health [12].

Montsko et al. [13] tested a wide range of wines to find out which type of wine is richest in polyphenols. The richest in *trans*-resveratrol were Pinot noir and St. Laurent red wines. For moderate daily consumption, the daily dosage of these two types of red wine that reduced the risk of cancer and cardiovascular diseases was 0.3 L for men and around 0.2 L for women. Pandey and Rizvi [14] reviewed the cardioprotective effects of resveratrol on myocardial cells against ischemia. Its role as a sirtuin activator, which detoxifies the body, was studied on reactive oxygen species (ROS). The results showed that resveratrol increased the cardioprotective effect against oxidative damage.

Due to its important bioactivities and the high popularity of red wine, resveratrol has spread amongst the public in the form of food supplements. Chinese laboratories studied the role of resveratrol in protecting endothelial function. The study reported a resveratrol dosage that protects blood vessels in vivo and in vitro. Nowadays, it is used as a food supplement for diabetic angiopathy prevention [15,16].

Das et al. [17] investigated the effects of resveratrol on cellular signalling networks that involved protein kinase C (PKC alpha) as a possible mechanism by which resveratrol affects many disease states. Resveratrol and its derivatives reduced PKC alpha activity. This strategy can be used to regulate diseases affected by protein kinase C. The main goal of the Mannari et al. [18] article was to investigate the cardio- and nephroprotective effects of resveratrol by modulating the nitric oxide (NO) system. Their research showed that one of the main actions of NO modulation is sirtuin regulation, especially by the SIRT1 protein. Resveratrol is a sirtuin activator. Final results showed that incubation of tubular cells with resveratrol increased SIRT1 expression as compared to the control group.

Ghanim et al. [19] studied the effect of resveratrol using a plant extract on heart and vascular diseases. The group with resveratrol intake on an empty stomach had no change in the concentrations of LDL (low density lipoprotein), HDL (high density lipoprotein), cholesterol, triglycerides, and leptin compared to the control group. This study pointed out the importance of an exact dose to ensure a definitive positive effect. If a consistent effective dosage was not used, a loss of antioxidant ability and nonspecific reactions with proteins occurred. Food supplements with resveratrol were mentioned in the review of Rahman et al. [20]. A wide range of beneficial effects of resveratrol on human health was described. The most significant results were reductions of cardiovascular diseases (hypertension, hypertrophy, coronary artery disease, and arteriosclerosis) in a group of people with regular moderate wine intake. The mental health of wine consumers was also better than the mental health of non-drinkers.

Romain et al. [21] investigated the effect of a grapevine shoot extract (Vineatrol 30) with a high concentration of resveratrol on the cardiovascular and hepatic systems in hamsters with induced arteriosclerosis. The extract increased antioxidant and anti-inflammatory activity and prevented aortic lipid deposition, which protects against infarction and other heart diseases. Mokni et al. [22] also studied the cardioprotective effects of resveratrol in rats. Data showed that resveratrol improved recovery of post-ischemic ventricular functions compared to control animals. Resveratrol also improved myocardial lipoperoxidation, myocardial free iron, and antioxidant enzyme activities.

The effect of certain doses of resveratrol on blood pressure, oxidative stress, and reactive oxygen species has been studied [19,23,24,25,26,27,28,29]. A summary of these studies is shown in Table 1.

Dos Santos et al. [30] studied resveratrol to evaluate its effects on calorimetric parameters (oxygen consumption, carbon dioxide production, respiratory quotient), antioxidants in the myocardium, and the energy metabolism of diabetic rats. The results showed that the cardioprotective effect of resveratrol may be mediated through its ability to normalize free fatty acid oxidation, enhance glucose utilization, and control the level of oxidative stress under diabetic conditions.

Semba et al. [31] demonstrated the possible effects of resveratrol on study participants, which included both men and women who were 65 years or older and from the Chianti area. Of the 639 participants, 174 developed cardiovascular disease and this occurrence was higher with a decreasing concentration of resveratrol. Urinary resveratrol metabolites demonstrated no substantial influence on longevity and lowering the risk of cardiovascular diseases when resveratrol intake was dietary. Menet et al. [32] showed a positive absorption of resveratrol and its metabolites in the plasma of non-human primates (*Microcebus murinus*), and implied a similarity with humans.

Another antioxidant present in red wine, as well as in tea, various fruits, vegetables, and seeds, is flavonoid rhamnetin. Park et al. [33] studied the effect of rhamnetin on cardiac myocytes under oxidative stress and showed the protective effects of rhamnetin against apoptosis in cardiomyoblasts by inhibiting free oxygen radicals.

Quintieri et al. [34] investigated malvidin (malvidin-3-0-glucoside or oenin), the most common wine anthocyanin, on cardiovascular function. The study results confirm the significant positive effects of malvidin, the most common polyphenol in red grape extracts, on cardioactivity by eliciting cardioprotection against ischemia/reperfusion damage. This major red wine anthocyanin, malvidin, was also closely studied by Bognar et al. [35] using cell exposure to malvidin. Malvidin reduced the course of chronic inflammation, inhibited phosphorylation activation, nuclear translocation, mitochondrial destabilization, prevented the formation of oxygen free radicals, and increased antioxidant activity.

**Figure 1:**
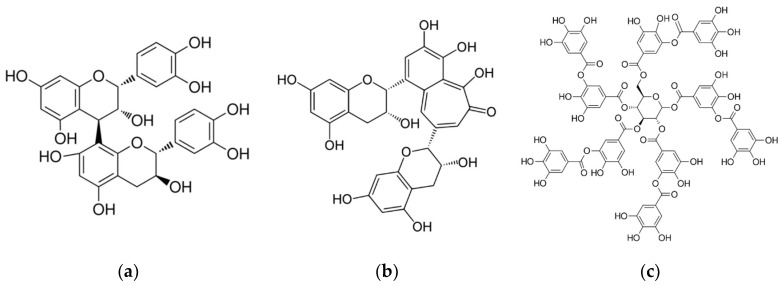
Tannins—(**a**) condensed (proanthocyanidins); (**b**) derived (theaflavin); and (**c**) hydrolysable (tannic acid).

An important polyphenol class in wine, from the grape skin, seeds, and stems, is the tannins. The main classes are condensed tannins (proanthocyanidins), created by the polymerization of flavan-3-ol monomers, and hydrolysable tannins (from oak barrels). Tannin structures are shown in Figure 1. 

Oenological processing can affect the final concentrations of these compounds. They are often present in red wine in levels of 300 mg/L [36]. Corder et al. [37] identified red wine procyanidins as the principal vasoactive polyphenols. Traditional wine production methods confirm that procyanidins are efficiently extracted during vinification. The oak barrels are used in viticulture to ripen red wine. Panchal et al. [38] studied the effects of a mixture of ellagitannins from oak bark (*Quercus petraea* L.) on cardiovascular, metabolic, and hepatic changes in spontaneously hypertensive rats and rats on a high-fat diet. The tannin extracts improved cardiovascular, metabolic, and hepatic function, which shows that ellagitannins from oak bark can enhance the positive effects of red wine.

Rossi et al. [39] studied the relationship of dietary antioxidants from beverages, such as wine, with acute myocardial infarction and its prevention. They found that the amount of antioxidants was inversely related to the risk of acute myocardial infarction. The authors encouraged moderate consumption of wine and high consumption of fruit and vegetables.

## 3. Alcohol and Wine Consumption and Their Impact on Human Health

The relationship between alcohol and wine consumption and human health effects has been studied by many researchers [40,41,42,43,44,45,46,47,48,49,50,51,52].

Elmadhun et al. [40] carried out an experimental study on pigs. Their findings suggested that, in moderate doses, ethanol directly promotes new vessel growth in nonischemic myocardium. Platisa et al. [41] have investigated the protective effect of a moderate consumption of red wine on the cardiovascular system. In separate experiments, volunteers drank 0.2 L of red wine and 0.2 L of a control alcohol drink (13.5% alcohol). The immediate effect of both drinks was equal and they increased systolic and diastolic blood pressure. However, after ten minutes, all measured data returned to normal. The prolonged effect of wine and the control drink was different as wine decreased blood pressure and reduced the complexity of the heart-interbeat interval and ventricular repolarization interval.

Matsumoto et al. [42] studied the positive effects of moderate alcohol intake on cardiovascular diseases in adult women and men. The results showed that consumption of 0.15 L of wine or 0.33 L of beer or 0.03 L of liquor lowered the risk of ischemic myocardium, cardiomyopathy, and overall mortality.

The study of Toth et al. [43] indicates the importance of a definitive alcohol dose for a positive effect. Using 39 healthy, non-smoking volunteers aged 18 to 40 years (one group drank water, second group drank 0.2 L of red wine each day for three weeks), they demonstrated the favourable hemorheological effects of red wine. After three weeks, red blood cell aggregation decreased in the group drinking the red wine. Red wine consumption increased red blood cell deformability at high shear stress. These results show that moderate red wine consumption has beneficial effects on haematological parameters, which are generators of cardiovascular diseases, especially coronary artery disease. Elmadhun et al. [44] studied the effects of low to moderate alcohol intake on the risk of developing cardiovascular diseases. They developed a clinically relevant animal model (pigs) for chronic myocardial ischemia to investigate the effects of moderate alcohol consumption on the myocardium. The results showed that alcohol consumption regulates apoptosis and promotes cell survival in ischemic and non-ischemic myocardium. Wine was recommended for its optimal amount of ethanol and other substances with cardioprotective effects. A dietary study of 108 patients with carotid atherosclerosis (65% on statin therapy) who did or did not drink red wine daily (0.1 L women, or 0.2 L men) was performed by Droste et al. [45]. The modified diet and physical exercise, as well as a daily glass of red wine, independently improved the LDL/HDL ratio in patients with arteriosclerosis, even for those on statin therapy.

Chu et al. [46] investigated the effects of two alcoholic beverages, red wine (Pinot noir) and vodka, on cardiovascular function in hypercholesterolemic swine. The goal was to improve a perfusion of ischemic myocardium. Daily intake of 0.375 L of wine or 0.1 L of vodka reduced the risk of cardiovascular diseases compared to normal adult swine.

The objective of the study of Yoo et al. [47] was to examine the health perceptions of red and white wine drinkers from Australia and Korea. Consumers prefer red wine, consuming this with an awareness of the health benefits and protection against myocardial infarction, cardiovascular diseases, and hypertension. The cardioprotective effect of moderate alcohol consumption was examined by Djoussé et al. [48]. The participants of this study were women, including both alcohol consumers and non-drinkers. The participants’ cardiovascular mortality and alcohol consumption rates were followed for 12 years. As compared with abstainers, alcohol drinkers, with an intake of 5 to 15 g per day, were associated with a 26% lower risk of cardiovascular disease, 35% lower risk of total mortality, and 51% lower risk of cardiovascular disease mortality, if the alcohol consumption was mostly red wine. Another study investigated the effects of regular alcohol consumption on the incidence of cardiovascular diseases and all-cause mortality. They studied the aspects of the overall alcohol-drinking pattern, all-cause mortality, and the risk of cancer and cardiovascular disease. The participants were more than 20,000 Mediterranean university graduates, surveyed every two years. The results showed a significantly lower incidence of death and cardiovascular diseases among wine drinkers than among beer or other alcohol drinkers. This outcome was associated with alcohol intake volume (men 20 g/day, women 10 g/day) in combination with polyphenol intake and the healthier lifestyle of wine-drinkers [49].

O′Keefe et al. [50] wanted to clarify the healthy intake of alcohol as heavy alcohol use is an important factor in the occurrence of reversible hypertension, nonischemic cardiomyopathy, atrial fibrillation, and risk of ischemic stroke. This study had 27,000 participants, including both men and women. They specified that regular low- to moderate-dose alcohol intake (0.1 L of wine per day for women and up to 0.2 L of wine per day for men) is associated with a decrease in the risk of adverse cardiovascular outcomes. The most suitable alcohol source seemed to be red wine.

During few last decades, the French paradox has been mentioned quite often. It means that French people have a relatively low incidence of coronary heart disease despite a diet that includes a high amount of saturated fat. Yamagata et al. [51] studied the preventive effects of polyphenols on cardiovascular diseases and their positive effects on the function of the endothelial cells. Observations of 129,000 adults showed a decreased risk of mortality from cardiovascular diseases in wine consumers compared to other alcohol drinkers. Sinkiewicz et al. [52] also reviewed the reason for the French paradox. Here, lower coronary artery disease occurrence was associated with consumption of wine rather than beer and other alcoholic beverages. People drinking three glasses of red wine every day had the lowest risk of cardiac diseases and mortality. Sufficient daily wine intake also decreased high blood pressure and the risk of myocardial infarction in men aged 65 years and older.

The Hispanic paradox is also being examined in the literature, as well as the French paradox. This anomaly was reviewed by Medina-Inojosa et al. [53]. Daily moderate wine consumption was considered the main reason for decreased cardiovascular diseases. A couple of Hispanic groups living in Europe, USA, and South America were observed and data about their lifestyle, foods, and health condition were collected. The results showed a lower occurrence of cardiovascular diseases compared to other non-Hispanic inhabitants.

Vilahur and Badimon [54] reviewed the cardioprotective effects of the Mediterranean diet on coronary artery disease and atherosclerosis. Their review confirmed that long-term, daily red wine consumption of 0.15 L for women and 0.45 L for men reduced inflammation and atherosclerosis, and improved lipid metabolism, antioxidant state, and endothelial function. Cioni et al. [55] compared relationships between life style, especially eating habits, and endothelial function and coronary artery disease. The study population was composed of 95 clinically stable females (47.9%) and males (42.1%) in primary prevention for cardiovascular disease. All participants underwent a medical questionnaire, clinical examination, and peripheral-arterial tonometry. The reactive hyperaemia index values were higher and the risk of endothelial dysfunction and cardiovascular diseases decreased for participants on the Mediterranean diet with moderate wine consumption. Giacosa et al. [56] describes a Mediterranean diet as being effective in cardiovascular prevention and mortality. This study showed that the Mediterranean diet is beneficial to health even among populations outside the Mediterranean basin.

Tognon et al. [57] studied Dutch inhabitants with different eating habits and the related risk of stroke, coronary heart disease, and cardiovascular mortality. The study had 1849 men and women as participants. The group with Mediterranean dietary eating habits was associated with a lower risk of coronary heart disease and cardiovascular mortality, but not of stroke. The group consuming wine for its alcohol intake was inversely associated with total mortality and with cardiovascular and myocardial infarction incidence and mortality. Stricker et al. [58] explored differences in 40,011 men and women in one group with high intakes of fish, high-fibre products, vegetables, and wine, and a second group, with a Western lifestyle (high consumption of French fries, fast food, low-fibre products, and other alcoholic drinks), for the occurrence of coronary heart disease and stroke. After 13 years of follow-up, 1843 coronary heart disease and 588 stroke cases were documented. The first group was associated with a reduced risk of coronary heart disease and stroke, and the second Western group was not related to any outcome.

Table 2 summarizes the benefits of wine or alcohol and their effects on human health.

## 4. Red Wine Effects on Humans Organs

The first organ that encounters the wine is the oral cavity followed by the esophagus.

Phytochemicals contained in wine, such as lignans, quercetin, and resveratrol, have estrogenic properties. Peterson et al. [59] reviewed several studies about lignans and confirmed a positive effect on cardiovascular diseases. Animal research suggests that they synergistically reduce the incidence of cancer. The protective effects of lignans on the development of human esophageal cancer have shown that these three phytochemicals can synergistically reduce esophageal cancer [60]. A serious disease of the esophagus is Barrett′s esophagus, a premalignant condition. Barrett′s esophagus is a complication of gastroesophageal reflux disease [61]. It was supposed that alcohol may increase the risk of Barrett′s esophagus through direct contact with esophageal mucosa. However, findings for this association are inconsistent. Inverse associations were observed for wine and beer consumption, and liquor consumption doubled the risk, but they were not statistically significant. Moreover, wine consumption has been associated with a moderately reduced risk of Barrett′s esophagus [62,63].

The stomach follows the esophagus. The gastric mucosa can be colonized by *Heliobacter pylori*, a gram-negative bacterium. Alcoholic beverages, especially red wine, probably have antibacterial activity against *Helicobacter pylori* and a protective effect against the ulcerogenic effect of ethanol on gastric mucosa. Flavonoids are responsible for these beneficial effects [64]. The consumption of 0.1 L of red wine has the potential to reduce the risk of gastric cancer. A statistical analysis by Barstad et al. [65] was carried out using a linear trend test. At the end of the study, it was suggested that daily intake of wine can prevent the development of stomach cancer.

The intestines are important organs in the gastrointestinal tract of the digestive system, and their cancers are the third most common cancer in men and women, accounting for 9% of total cancer deaths [66]. Polyphenols contained in wine can prevent or delay the progression of inflammatory bowel disease of the colon and small intestine. Wine polyphenols act as free radical scavengers and modulators of specific genes and can act as probiotics and antimicrobials. Therefore, they could be used for the prevention or treatment of inflammatory intestinal diseases [67,68]. These theses were verified by an experiment on intestinal tumor progression in rats by administering 0.2 mg of *trans*-resveratrol in their drinking water over 100 days. A significant, visible decrease in the tumors was observed after a daily dosing of 8 mg of *trans*-resveratrol. The results showed that this polyphenol can suppress the progression of the malignant phase of intestinal cancer [69].

The next organ influenced by wine consumption is the liver. Hepatocellular carcinoma, also called primary cancer, is the sixth most common cancer in the world. Bishayee et al. [70], in his review, found resveratrol prevents and controls hepatocellular carcinoma. Resveratrol inhibited carcinogenesis, with a pleiotropic effect. Carbo et al. [71] investigated the antitumor potential of resveratrol in animal models of liver cancer. They showed that the use of resveratrol has not only a chemopreventive effect, but it is also effective as a chemotherapeutic agent. Following administration of resveratrol to rats inoculated with rapidly growing hepatoma cells, resveratrol caused a significant reduction in the tumor cell number. Oral administration of 20 mg of resveratrol daily for six weeks can significantly prevent the loss of liver weight and inhibit serum alanine aminotransferase, aspartate aminotransferase, alkaline phosphatase, and bilirubin levels. Resveratrol had in vivo hepatoprotective effects against antifibrogenic dimethylnitrosamine-induced liver injury, suggesting that it may be useful in preventing the development of hepatic fibrosis [72].

Pancreatic cancer has few early symptoms and is usually diagnosed in the later stages. Alcohol has been promoted as a supporter of pancreatic cancer. Recent epidemiological studies show contradictory results regarding alcohol intake and pancreatic cancer. Resveratrol binds directly to leukotriene A(4) hydrolase in vitro and in cells, and suppresses the proliferation of and anchorage-independent pancreatic cancer growth by inhibiting leukotriene B(4) production and leukotriene B(4) receptor 1 expression. Resveratrol has a relatively stronger inhibitory effect than bestatin, an inhibitor of leukotriene A(4) hydrolase activity. It is important that resveratrol inhibits tumor formation in the xenograft model of human pancreatic cancer cells [73].

Table 3 summarizes the information on human organs and the effects of various components of wine on various diseases.

## 5. Biological Mechanisms of Human Body Influenced by Wine Consumption

### 5.1. Lipid Metabolism

Da Luz et al. [74] investigated the health benefits of moderate red wine consumption, with a focus on coronary vasculature. The study included 101 alcohol drinkers aged around 60 years and 104 abstainers. The results showed that regular red wine drinkers have similar coronary plaque and endothelial function, higher HDL cholesterol level and lower occurrence of coronary lesions compared to abstainers.

Janega et al. [75] analysed the effect of alcohol-free Alibernet red wine extract on nitric oxide synthase activity and pro-inflammatory markers that affect genes significant for immunity and inflammation in rats. Alibernet red wine extract treatment did not affect total and endothelial NO synthase activity, but it was able to decrease pro-inflammatory marker activity and inducible NO synthase expression in both the left ventricle and aorta. Calorie restriction in combination with an exact resveratrol dosage was associated with cardioprotective function against doxorubicin-induced damage in 26-month-old rats. This was also observed by Dutta et al. [76] in the hearts of 26-month-old rats. They studied resveratrol as a possible autophagy inductor. Autophagy is a cellular self-digestion process whereby cells degrade dysfunctional proteins and organelles. Other beneficial results were the vasodilatory, anti-inflammatory and antioxidative effects.

Lee et al. [77] checked the effects of non-alcoholic red wine concentrate on cholesterol level and related occurrence of cardiovascular diseases using in vitro and in vivo models. Regular moderate consumption reduced the risk of cardiovascular diseases and increased antioxidant activity. The study demonstrated the reduction of plasma cholesterol, LDL cholesterol and total cholesterol in an animal model, as well as a decrease in intracellular total cholesterol and triglyceride in vitro. HDL cholesterol remained unchanged.

Karadeniz et al. [78] concentrated their attention on possible solutions to lower high obesity and heart diseases. A direct relationship between obesity, oxidative stress markers and susceptibility of LDL to oxidation was demonstrated. As expected the levels of total cholesterol, triglycerides and LDL cholesterol were higher in obese patients, but following wine consumption, antioxidant and paraoxonase activities increased, which led to a decreased LDL cholesterol level.

Xiang et al. [79] conducted a study on the effect of red wine consumption and its beneficial effects on reducing the risk of heart diseases and lifespan extension attributed to its high antioxidant content. The aim of the article was to study relative contribution of wine polyphenols on health. They found that anti-radical activity correlates with total amount of polyphenols.

The paragraph dealing with lipid metabolism is summarized in the Table 4.

### 5.2. Diabetes Mellitus

Da Luz et al. [74] investigated the health benefits of moderate red wine consumption, with a focus on glucose levels and diabetes. The study included 101 alcohol drinkers aged around 60 years and 104 abstainers. The results showed that regular red wine drinkers have lower glucose levels and a lower occurrence of diabetes compared to abstainers.

Bresciani et al. [80] conducted a study with 73 male rats of 13-weeks old. The groups with no resveratrol intake and the lowest dosage had the highest glucose level increases. The group with the highest resveratrol dosage had decreased glucose levels after three weeks. The highest dosage intake led to the recovery of hemodynamic performance. The lower dosage was less effective.

Chiva-Blanch et al. [81] studied the benefits of moderate consumption of alcohol (red wine, dealcoholized red wine, and gin) on glucose metabolism. Moderate alcohol intake, either red wine (30 g of alcohol per day), the same amount of dealcoholized red wine, and gin (same amount of alcohol), consumed for four week periods demonstrated that red wine and dealcoholized red wine consumption decreased plasma insulin and the homeostasis model assessment of insulin resistance, therefore, it was concluded that red wine is protective against type 2 diabetes in association with an improved insulin sensitivity.

Blomster et al. [82] had similar results in patients with type 2 diabetes. The prevalence of type 2 diabetes is increasing and is influenced by lifestyle factors, such as high-calorie diet consumption, sedentary behaviour, and the resultant increase in excess weight. The aim of this study was to establish the effects of moderate alcohol consumption on cardiovascular disease in diabetic patients. Compared with patients who reported no alcohol consumption, those who reported moderate consumption had fewer cardiovascular events and a lower all-cause mortality. The benefits were particularly evident in participants who predominantly drank wine.

This section dealing with diabetes mellitus is summarized in the Table 5.

### 5.3. Oxidative Stress

Oxidative stress is an important component of various diseases related to the production of reactive oxygen species, cardiovascular risk factors, and cellular dysfunction.

Chiva-Blanch et al. [83] evaluated the effects of alcoholic and non-alcoholic red wine and gin consumption in 67 men with high cardiovascular risk. They randomized them into groups with a dose of either red wine or gin, both with the same alcohol content. Dealcoholized red wine decreased systolic and diastolic blood pressure, which were correlated with increases in plasma NO. Red wine prevented fat microparticle sedimentation after a high-fat meal consumption, which could lead to endothelial dysfunction and cardiovascular events. This effect was also shown by Bulut et al. [84]. Ten healthy males consumed French fries and pork sausages, both high in fat, once a week for four weeks. They had mineral water, Coke, red wine, and liquor as beverages. It was discovered that a single, high-fat meal deteriorates endothelial function, which was associated with a significant increase in circulating microparticles. These negative effects were decreased with red wine.

Oxidative stress plays an important role in arteriosclerosis development. Toth et al. [85] studied hemorheological parameters in over 1000 patients diagnosed with various forms of ischemic heart disease during the past decades. In their in vitro and in vivo study, they investigated the effects of red wine on hemorheological parameters, which play a critical role in the pathogenesis of myocardial ischemia. The results showed that moderate red wine consumption has beneficial effects on these parameters, which may explain the French paradox.

Dillenburg et al. [86] evaluated changes in cardiovascular parameters following resveratrol and polyphenol intake in male Wistar rats. The results indicated the beneficial effects of resveratrol and grape juice on endothelial nitric oxide synthase, as well as a decrease of vagal modulation and an increase of the alpha index in the resveratrol group. Resveratrol influenced not only cardiac, but also vascular, autonomic modulation. Both resveratrol and grape juice showed beneficial effects, lowering the risk of possible heart damage during hypertension and eliminating hypertension.

Levantesi et al. [87] evaluated the association of wine intake with an incidence of cardiovascular events and total mortality after myocardial infarction. The participants included 11,248 Italian patients who had a recent myocardial infarction, and wine consumption was divided into three groups: Never/almost never, up to 0.5 L per day, and over this amount. Moderate wine intake was associated with a reduced risk of cardiovascular events as compared to abstainers. The risk of total mortality was lower in patients with regular wine intake, but only up to a daily wine consumption of 0.5 L. The overall results showed that moderate wine consumption in patients with established heart disease led to a decrease in other cardiovascular events.

The protective properties of red wine in relation to chronic diseases are summarized in Table 6.

## 6. Conclusions

After studying the recent available clinical trials, it is evident that not only healthy food, but also moderate consumption of wine, has a link to cancer prevention. Biological mechanisms for oncological prevention are associated with the consumption of antioxidants and polyphenols that are contained in fruits, their products, such as wine, and vegetables. A moderate consumption of wine is recommended daily, mainly with food [56].

Epidemiological studies have shown that five to seven portions of fresh fruit and vegetables and two glasses of wine a day can lead to a longer and healthier life [87]. The beneficial effect of wine is attributed mainly to its antioxidant properties of the large number and amount of polyphenolic compounds present in red wine [88].

It is recommended that a moderate, optional, daily alcohol consumption is about 15 and 30 g of alcohol for women and men, respectively. The highest tolerated dose ranges up to 36 g per day for healthy women and up to 60 g per day for healthy men.

Wine polyphenols could be effective in preventing cardiovascular diseases (CVD). Clinical studies show that CVD can be influenced by moderate wine consumption. These theses are supported by many scientists and by scientific tests and clinical studies, but some dismiss the beneficial effects of resveratrol. According to the above-mentioned studies, it is possible to strengthen the effect of resveratrol through a well-balanced diet, such as a form of the “Mediterranean diet”, that includes red wine, fish, fruits, and vegetables, and foods high in dietary fibre, vitamins, and minerals in the diet. According to the research, it can be assumed that the substances contained in red wine and their joint action, when they complement each other, produce the desired effect, namely protection against cardiovascular diseases. One mechanism of action for resveratrol is reducing damage to the cardiovascular system due to oxidative stress by neutralizing free oxygen radicals and reactive nitrogenous radicals. Compared with other antioxidants, resveratrol can penetrate the brain-brain barrier and, thus, protect the brain and nerve cells. Resveratrol also reduces platelet aggregation and, thus, counteracts the formation of blood clots or thrombi. The positive effects of wine consumption may fluctuate depending on the individual substances, especially due to their absorption, the perception of biologically active substances, and other factors. Thus, this topic requires further careful research.

## Figures and Tables

**Table 1:** Summary of some studies with resveratrol dosage (mg) and its effect.

Participants	Condition	Resveratrol Dose (mg)	Duration	Downregulation Effect	Ref.
20 (undifferentiated sex)	healthy	40	6 weeks	Reactive oxygen species (ROS)	[19]
10 (4 men, 6 women)	healthy	100	1 week	ROS, Toll-like receptor 4	[23]
19 (men)	diabetic	10	1 month	ROS	[24]
11 (men)	obese	300	1 month	ROS, glucose, insulin	[25]
50 (undifferentiated sex)	smokers	500	1 month	ROS, C-reactive protein	[26]
62 (undifferentiated sex)	healthy	250	3 month	Systolic BP, cholesterol	[27]
10 (men)	obese	150	1 month	Postprandial glucagon resp.	[28]
24 (undifferentiated sex)	diabetic	100	2 month	Reduction of foot ulcer size	[29]

**Table 2:** Summary of recommended doses of wine or other alcohol and their effects on human health.

Recommendation	Kind of Alcohol	Effect	Refs.
0.2 L	Red wine	Increased systolic and diastolic blood pressure, hypertension	[41,47]
0.15 L of wine or 0.33 L of beer or 0.03 L of liquor		Lowered the risk of ischemic myocardium, cardiomyopathy and overall mortality	[42]
0.2 L	Red wine	Beneficial effects on coronary artery disease, cardioprotective effect	[43,48]
0.1 L women or 0.2 L men of red wine		Improved independently the LDL/HDL ratio in patients with arteriosclerosis	[45,50]
0.375 L of wine or 0.1 L of vodka		Reduce the risk of cardiovascular diseases	[46]
0.15 L for women, 0.45 L for men of red wine		Reduced inflammation, atherosclerosis, improved lipid metabolism, antioxidant state, and endothelial function	[54]

**Table 3:** Overview of human organs, their diseases, and the effects of wine components.

Organ	Disease	Substance and Dosage	Effect	Refs.
Esophagus	Cancer Barrett’s esophagus	Phytochemicals (lignans, quercetin, resveratrol) in wine	Synergistically reduces esophageal cancer, moderately reduced risk of Barrett’s esophagus	[59,60,61,62,63]
Stomach	*Helicobacter pylori* Cancer	Flavonoids, ethanol, alcoholic beverages, especially red wine 0.1 L/day of red wine	Antibacterial activity, protective effect against the ulcerogenic effect of ethanol on gastric mucosa	[64,65]
Intestines	Cancer IBD	Wine polyphenols, probiotics, and antimicrobials 8 mg of *trans*-resveratrol	Reduced progression of malignant phase of cancer, prevent or delay progression of inflammatory bowel disease of colon and small intestine	[66,67,68,69]
Liver	Hepatocellular carcinoma	Resveratrol 20 mg resveratrol daily	Inhibited carcinogenesis with a pleiotropic effect, chemopreventive effect, in vivo hepatoprotective effects	[70,71,72]
Pancreas	Cancer	Resveratrol	Suppresses proliferation of anchorage-independent growth by inhibiting leukotriene B(4) production, leukotriene B(4) receptor 1 expression	[73]

**Table 4:** Randomized controlled trial of lipid metabolism.

Study Model	Outcome	Study	Effect	Refs.
In vivo	Levels of total cholesterol, triglycerides and LDL cholesterol	Effects of non-alcoholic red wine concentrate on cholesterol level and related occurrence of cardiovascular diseases	Antioxidant and paraoxonase activities increased, decrease in intracellular total cholesterol and triglyceride	[77,78]
In vitro				
Sewer rats	Autophagy	Resveratrol as a possible autophagy inductor	Vasodilatory, anti-inflammatory and antioxidative effects	[76]
Rats	Endothelial NO	Effect of alcohol-free Alibernet red wine extract on NO synthase activity and pro-inflammatory markers	Decrease pro-inflammatory marker activity and inducible NO synthase expression in both the left ventricle and aorta	[75]
Humans	LDL, HDL, coronary plaque and endothelial function	Health benefits of moderate red wine consumption	Higher HDL cholesterol level and lower occurrence of coronary lesions compared to abstainers	[74]

**Table 5:** Randomized controlled trial of diabetes mellitus.

Study Model	Outcome	Study	Effect	Ref.
Drinkers vs.	Glucose level and diabetes	Health benefits of moderate red wine consumption	Drinkers have lower glucose levels and a lower occurrence of diabetes compared to abstainers	[74]
Abstainer				
Rats	Glucose level	Dosage of resveratrol	Highest resveratrol dosage decreased glucose levels	[80]
Men	Glucose metabolism	Benefits of moderate consumption of alcohol (red wine, dealcoholized red wine, and gin)	Decreased plasma insulin and the homeostasis model assessment of insulin resistance	[81]
Type 2 diabetics	CVD	Establish the effects of moderate alcohol consumption on cardiovascular disease in diabetic patients	Moderate consumption had fewer cardiovascular events and lower all-cause mortality	[82]

**Table 6:** Summary of the protective effects of red wine in relation to chronic diseases.

Effect	Mechanism of Action	Study Model	Refs.
Anti-inflammatory	Prevented aortic lipid deposition, inhibited phosphorylation activation, nuclear translocation, mitochondrial destabilization, prevented the formation of oxygen free radicals, increased antioxidant activity	Hamsters, human cells, humans	[21,26,38,54,67]
	Inhibition of nitric oxide production, progression of the malignant phase of intestinal cancer	Rats	[69,75]
Antidiabetic	Prevention of diabetes	In vivo and in vitro models, humans	[10,18,47]
	Enhanced utilization of glucose and controlled the level of oxidative stress under diabetic conditions	Rats	[26,30]
	Decreased the glucose level	Rats	[15,80]
Cardioprotective	Reduced the risk of cardiovascular diseases	Humans, swine, rats	[6,44]
	Decreased the oxidative stress	Human cells, humans, rats, hamsters	[79,83,84,85,86,87]
	Hypertension, hypertrophy, coronary artery disease, and arteriosclerosis	Humans	[21,22,31,43]

## References

[1] Lukacs P. (2012). Inventing Wine: A New History of One of the World’s Most Ancient Pleasures.

[2] Snopek L., Mlček J., Fic V., Hlaváčová I., Škrovánková S., Fišera M., Velichová H., Ondrášová M. (2018). Interaction of polyphenols and wine antioxidants with its Sulfur dioxide preservative. Potravin. Slovak J. Food Sci..

[3] Teissedre P.L., Stockley C., Boban M., Ruf J.C., Alba M.O., Gambert P., Flesh M. (2018). The effects of wine consumption on cardiovascular disease and associated risk factors: A narrative review. OENO ONE.

[4] Klatsky A.L. (2009). Alcohol and cardiovascular diseases. Expert Rev. Cardiovasc. Ther..

[5] Cavallini G., Straniero S., Donati A., Bergamini E. (2016). Resveratrol requires red wine polyphenols for optimum antioxidant activity. J. Nutr. Health Aging.

[6] Singleton V.L., Rossi J.A. (1965). Colorimetry of total phenolics with phosphomolybdic-phosphotungstic acid reagents. Am. J. Enol. Vitic..

[7] Del Pino-García R., González-SanJosé M.L., Rivero-Pérez M.D., García-Lomillo J., Muñiz P. (2017). The effects of heat treatment on the phenolic composition and antioxidant capacity of red wine pomace seasonings. Food Chem..

[8] Kim J.H., Auger C., Kurita I., Anselm E., Rivoarilala L.O., Lee H.J., Schini-Kerth V.B. (2013). Aronia melanocarpa juice, a rich source of polyphenols, induces endothelium-dependent relaxations in porcine coronary arteries via the redox-sensitive activation of endothelial nitric oxide synthase. Nitric Oxide-Biol. Chem..

[9] Schini-Kerth V.B., Auger C., Étienne-Selloum N., Chataigneau T. (2010). Polyphenol-Induced Endothelium-Dependent Relaxations: Role of NO and EDHF. Cardiovasc. Pharmacol. Endothel. Control.

[10] Qureshi S.A., Lund A.C., Veierød M.B., Carlsen M.H., Blomhoff R., Andersen L.F., Ursin G. (2014). Food items contributing most to variation in antioxidant intake; a cross-sectional study among Norwegian women. BMC Public Health.

[11] Tomé-Carneiro J., Gonzálvez M., Larrosa M., Yáñez-Gascón M.J., García-Almagro F.J., Ruiz-Ros J.A., Espín J.C. (2013). Resveratrol in primary and secondary prevention of cardiovascular disease: A dietary and clinical perspective. Ann. N. Y. Acad. Sci..

[12] Tomé-Carneiro J., Larrosa M., González-Sarrías A., Tomas-Barberan F., Teresa Garcia-Conesa M., Carlos Espin J. (2013). Resveratrol and Clinical Trials: The Crossroad from In Vitro Studies to Human Evidence. Curr. Pharm. Des..

[13] Montsko G., Ohmacht R., Mark L. (2010). *trans*-Resveratrol and *trans*-Piceid Content of Hungarian Wines. Chromatographia.

[14] Pandey K.B., Rizvi S.I. (2011). Anti-oxidative action of resveratrol: Implications for human health. Arab. J. Chem..

[15] Peng X.L., Qu W., Wang L.Z., Huang B.Q., Ying C.J., Sun X.F., Hao L.P. (2014). Resveratrol Ameliorates High Glucose and High-Fat/Sucrose Diet-Induced Vascular Hyperpermeability Involving Cav-1/eNOS Regulation. PLoS ONE.

[16] Oh W.Y., Shahidi F. (2018). Antioxidant activity of resveratrol ester derivatives in food and biological model systems. Food Chem..

[17] Das J., Pany S., Majhi A. (2011). Chemical modifications of resveratrol for improved protein kinase C alpha activity. Bioorgan. Med. Chem..

[18] Mannari C., Bertelli A.A.E., Stiaccini G., Giovannini L. (2010). Wine, sirtuins and nephroprotection: Not only resveratrol. Med. Hypotheses.

[19] Ghanim H., Sia C.L., Abuaysheh S., Korzeniewski K., Patnaik P., Marumganti A., Dandona P. (2010). An antiinflammatory and reactive oxygen species suppressive effects of an extract of *Polygonum cuspidatum* containing resveratrol. J. Clin. Endocrinol. Metab..

[20] Rahman M.M., Bak I., Das D.K. (2010). Effectiveness of Resveratrol against Cardiovascular Disease. Mini-Rev. Org. Chem..

[21] Romain C., Gaillet S., Carillon J., Vidé J., Ramos J., Izard J.C., Rouanet J.M. (2012). Vineatrol and Cardiovascular Disease: Beneficial Effects of a Vine-Shoot Phenolic Extract in a Hamster Atherosclerosis Model. J. Agric. Food Chem..

[22] Mokni M., Hamlaoui S., Karkouch I., Amri M., Marzouki L., Limam F., Aouani E. (2013). Resveratrol Provides Cardioprotection after Ischemia/reperfusion Injury via Modulation of Antioxidant Enzyme Activities. Iran. J. Pharm. Res..

[23] Ghanim H., Sia C.L., Korzeniewski K., Lohano T., Abuaysheh S., Marumganti A., Dandona P. (2011). A resveratrol and polyphenol preparation suppresses oxidative and inflammatory stress response to a high-fat, high carbohydrate meal. J. Clin. Endocrinol. Metab..

[24] Brasnyó P., Molnár G.A., Mohás M., Markó L., Laczy B., Cseh J., Mészáros L.G. (2011). Resveratrol improves insulin sensitivity, reduces oxidative stress and activates the Akt pathway in type 2 diabetic patients. Br. J. Nutr..

[25] Timmers S., Konings E., Bilet L., Houtkooper R.H., van de Weijer T., Goossens G.H., Moonen-Kornips E. (2011). Calorie restriction-like effects of 30 days of resveratrol supplementation on energy metabolism and metabolic profile in obese humans. Cell Metab..

[26] Bo S., Ciccone G., Castiglione A., Gambino R., De Michieli F., Villois P., Cassader M. (2013). Anti-inflammatory and antioxidant effects of resveratrol in healthy smokers a randomized, double-blind, placebo controlled, cross-over trial. Curr. Med. Chem..

[27] Bhatt J.K., Thomas S., Nanjan M.J. (2012). Resveratrol supplementation improves glycemic control in type 2 diabetes mellitus. Nutr. Res..

[28] Knop F.K., Konings E., Timmers S., Schrauwen P., Holst J.J., Blaak E.E. (2013). Thirty days of resveratrol supplementation does not affect postprandial incretin hormone responses, but suppresses postprandial glucagon in obese subjects. Diabet. Med..

[29] Bashmakov Y.K., Assaad-Khalil S.H., Abou Seif M., Udumyan R., Megallaa M., Rohoma K.H., Petyaev I.M. (2014). Resveratrol promotes foot ulcer size reduction in type 2 diabetes patients. ISRN Endocrinol..

[30] Dos Santos K.C., Braga C.P., Barbanera P.O., Seiva F.R.F., Junior A.F., Fernandes A.A.H. (2014). Cardiac Energy Metabolism and Oxidative Stress Biomarkers in Diabetic Rat Treated with Resveratrol. PLoS ONE.

[31] Semba R.D., Ferrucci L., Bartali B., Urpí-Sarda M., Zamora-Ros R., Sun K., Andres-Lacueva C. (2014). Resveratrol Levels and All-Cause Mortality in Older Community-Dwelling Adults. JAMA Int. Med..

[32] Menet M.C., Marchal J., Dal-Pan A., Taghi M., Nivet-Antoine V., Dargère D., Cottart C.H. (2014). Resveratrol Metabolism in a Non-Human Primate, the Grey Mouse Lemur (*Microcebus murinus*), Using Ultra-High-Performance Liquid Chromatography–Quadrupole Time of Flight. PLoS ONE.

[33] Park E.S., Kang J.C., Jang Y.C., Park J.S., Jang S.Y., Kim D.E., Shin H.S. (2014). Cardioprotective effects of rhamnetin in H9c2 cardiomyoblast cells under H_2_O_2_-induced apoptosis. J. Ethnopharmacol..

[34] Quintieri A.M., Baldino N., Filice E., Seta L., Vitetti A., Tota B., Angelone T. (2013). Malvidin, a red wine polyphenol, modulates mammalian myocardial and coronary performance and protects the heart against ischemia/reperfusion injury. J. Nutr. Biochem..

[35] Bognar E., Sarszegi Z., Szabo A., Debreceni B., Kalman N., Tucsek Z., Gallyas F. (2013). Antioxidant and Anti-Inflammatory Effects in RAW264.7 Macrophages of Malvidin, a Major Red Wine Polyphenol. PLoS ONE.

[36] Gu L., Kelm M.A., Hammerstone J.F., Beecher G., Holden J., Haytowitz D., Prior R.L. (2004). Concentrations of Proanthocyanidins in Common Foods and Estimations of Normal Consumption. J. Nutr..

[37] Corder R., Mullen W., Khan N.Q., Marks S.C., Wood E.G., Carrier M.J., Crozier A. (2006). Oenology: Red wine procyanidins and vascular health. Nature.

[38] Panchal S.K., Brown L. (2013). Cardioprotective and hepatoprotective effects of ellagitannins from European oak bark (*Quercus petraea* L.) extract in rats. Eur. J. Nutr..

[39] Rossi M., Praud D., Compagnoni M.M., Bellocco R., Serafini M., Parpinel M., Tavani A. (2014). Dietary non-enzymatic antioxidant capacity and the risk of myocardial infarction: A case-control study in Italy. Nutr. Metab. Cardiovasc. Dis..

[40] Elmadhun N.Y., Sabe A.A., Lassaletta A.D., Sellke F.W. (2015). Ethanol promotes new vessel growth in remote nonischemic myocardium. J. Surg. Res..

[41] Platiša M.M., Gal V., Nestorović Z., Gojković-Bukarica L. (2014). Quantification of the acute effect of a low dose of red wine by nonlinear measures of RR and QT interval series in healthy subjects. Comput. Biol. Med..

[42] Matsumoto C., Miedema M.D., Ofman P., Gaziano J.M., Sesso H.D. (2014). An Expanding Knowledge of the Mechanisms and Effects of Alcohol Consumption on Cardiovascular Disease. J. Cardiopulm. Rehabil. Prev..

[43] Toth A., Sandor B., Papp J., Rabai M., Botor D., Horvath Z., Czopf L. (2014). Moderate red wine consumption improves hemorheological parameters in healthy volunteers. Clin. Hemorheol. Microcirc..

[44] Elmadhun N.Y., Sabe A.A., Lassaletta A.D., Sellke F.W. (2014). Alcohol Consumption Mitigates Apoptosis and Mammalian Target of Rapamycin Signaling in Myocardium. J. Am. Coll. Surg..

[45] Droste D.W., Iliescu C., Vaillant M., Gantenbein M., De Bremaeker N., Lieunard C., Gilson G. (2013). A daily glass of red wine associated with lifestyle changes independently improves blood lipids in patients with carotid arteriosclerosis: Results from a randomized controlled trial. Nutr. J..

[46] Chu L.M., Lassaletta A.D., Robich M.P., Liu Y., Burgess T., Laham R.J., Sellke F.W. (2012). Effects of Red Wine and Vodka on Collateral-Dependent Perfusion and Cardiovascular Function in Hypercholesterolemic Swine. Circulation.

[47] Yoo Y.J., Saliba A.J., MacDonald J.B., Prenzler P.D., Ryan D. (2013). A cross-cultural study of wine consumers with respect to health benefits of wine. Food Qual. Prefer..

[48] Djoussé L., Lee I.-M., Buring J.E., Gaziano J.M. (2009). Alcohol Consumption and Risk of Cardiovascular Disease and Mortality in Women: Potential Mediating Mechanisms. Circulation.

[49] Gea A., Bes-Rastrollo M., Toledo E., Garcia-Lopez M., Beunza J.J., Estruch R., Martinez-Gonzalez M.A. (2014). Mediterranean alcohol-drinking pattern and mortality in the SUN (Seguimiento Universidad de Navarra) Project: A prospective cohort study. Br. J. Nutr..

[50] O’Keefe J.H., Bhatti S.K., Bajwa A., DiNicolantonio J.J., Lavie C.J. (2014). Alcohol and Cardiovascular Health: The Dose Makes the Poison…or the Remedy. Mayo Clin. Proc..

[51] Yamagata K., Tagami M., Yamori Y. (2015). Dietary polyphenols regulate endothelial function and prevent cardiovascular disease. Nutrition.

[52] Sinkiewicz W., Weglarz M., Chudzinska M. (2014). Wine, alcohol and cardiovascular diseases. Kardiol. Polska.

[53] Medina-Inojosa J., Jean N., Cortes-Bergoderi M., Lopez-Jimenez F. (2014). The Hispanic Paradox in Cardiovascular Disease and Total Mortality. Prog. Cardiovasc. Dis..

[54] Vilahur G., Badimon L. (2013). Antiplatelet properties of natural products. Vasc. Pharmacol..

[55] Cioni G., Boddi M., Fatini C., Romagnuolo I., Casini A., Gensini G.F., Sofi F. (2013). Peripheral-Arterial Tonometry for Assessing Endothelial Function in Relation to Dietary Habits. J. Investig. Med..

[56] Giacosa A., Barale R., Bavaresco L., Gatenby P., Gerbi V., Janssens J., Morazzoni P. (2013). Cancer prevention in Europe: The Mediterranean diet as a protective choice. Eur. J. Cancer Prev..

[57] Tognon G., Lissner L., Sæbye D., Walker K.Z., Heitmann B.L. (2014). The Mediterranean diet in relation to mortality and CVD: A Danish cohort study. Br. J. Nutr..

[58] Stricker M.D., Onland-Moret N.C., Boer J.M.A., Van Der Schouw Y.T., Verschuren W.M.M., May A.M., Beulens J.W.J. (2013). Dietary patterns derived from principal component- and k-means cluster analysis: Long-term association with coronary heart disease and stroke. Nutr. Metab. Cardiovasc. Dis..

[59] Peterson J., Dwyer J., Adlercreutz H., Scalbert A., Jacques P., McCullough M.L. (2010). Dietary lignans: Physiology and potential for cardiovascular disease risk reduction. Nutr. Rev..

[60] Lin Y., Yngve A., Lagergren J., Lu Y. (2014). A dietary pattern rich in lignans, quercetin and resveratrol decreases the risk of oesophageal cancer. Br. J. Nutr..

[61] Bremholm L., Funch-Jensen P., Eriksen J., Hendel L., Havelund T., Matzen P. (2012). Barrett’s esophagus. Diagnosis, follow-up and treatment. Dan. Med. J..

[62] Thrift A.P., Cook M.B., Vaughan T.L., Anderson L.A., Murray L.J., Whiteman D.C., Corley D.A. (2014). Alcohol and the Risk of Barrett’s Esophagus: A Pooled Analysis from the International BEACON Consortium. Am. J. Gastroenterol..

[63] Thrift A.P., Pandeya N., Smith K.J., Mallitt K.A., Green A.C., Webb P.M., Whiteman D.C. (2011). Lifetime Alcohol Consumption and Risk of Barrett’s Esophagus. Am. J. Gastroenterol..

[64] Kasicka-Jonderko A. (2012). Alcohol and the digestive system—Should it always be blamed?. Prz. Gastroenterol..

[65] Barstad B., Sørensen T.I.A., Tjønneland A., Johansen D., Becker U., Andersen I.B., Grønbæk M. (2005). Intake of wine, beer and spirits and risk of gastric cancer. Eur. J. Cancer Prev..

[66] Rosen L., Rosen G. (2008). Cancer Facts and Figures.

[67] Biasi F., Deiana M., Guina T., Gamba P., Leonarduzzi G., Poli G. (2014). Wine consumption and intestinal redox homeostasis. Redox Biol..

[68] Seeram N.P., Aviram M., Zhang Y., Henning S.M., Feng L., Dreher M., Heber D. (2008). Comparison of antioxidant potency of commonly consumed polyphenol-rich beverages in the United States. J. Agric. Food Chem..

[69] Tessitore L., Davit A., Sarotto I., Caderni G. (2000). Resveratrol depresses the growth of colorectal aberrant crypt foci by affecting *bax* and *p*21^CIP^ expression. Carcinogenesis.

[70] Bishayee A., Politis T., Darvesh A.S. (2010). Resveratrol in the chemoprevention and treatment of hepatocellular carcinoma. Cancer Treat. Rev..

[71] Carbó N., Costelli P., Baccino F.M., López-Soriano F.J., Argilés J.M. (1999). Resveratrol, a natural product present in wine, decreases tumour growth in a rat tumour model. Biochem. Biophys. Res. Commun..

[72] Lee E.S., Shin M.O., Yoon S., Moon J.O. (2010). Resveratrol Inhibits Dimethylnitrosamine-Induced Hepatic Fibrosis in Rats. Arch. Pharm. Res..

[73] Oi N., Jeong C.H., Nadas J., Cho Y.Y., Pugliese A., Bode A.M., Dong Z. (2010). Resveratrol, a Red Wine Polyphenol, Suppresses Pancreatic Cancer by Inhibiting Leukotriene A_4_ Hydrolase. Cancer Res..

[74] Da Luz P.L., Coimbra S., Favarato D., Albuquerque C., Mochiduky R.I., Rochitte C.E., Laurindo F.R. (2014). Coronary artery plaque burden and calcium scores in healthy men adhering to long-term wine drinking or alcohol abstinence. Braz. J. Med. Biol. Res..

[75] Janega P., Klimentová J., Barta A., Kovácsová M., Vranková S., Cebová M., Pechánová O. (2014). Red wine extract decreases pro-inflammatory markers, nuclear factor-kappa B and inducible NOS, in experimental metabolic syndrome. Food Funct..

[76] Dutta D., Xu J., Dirain M.L., Leeuwenburgh C. (2014). Calorie restriction combined with resveratrol induces autophagy and protects 26-month-old rat hearts from doxorubicin-induced toxicity. Free Radic. Biol. Med..

[77] Lee D.H., Choi S.S., Kim B.B., Kim S.Y., Kang B.S., Lee S.J., Park H.J. (2013). Effect of alcohol-free red wine concentrates on cholesterol homeostasis: An in vitro and in vivo study. Process Biochem..

[78] Karadeniz M., Akçay Y.D., Yıldırım H.K., Yılmaz C., Sözmen E.Y. (2014). Effect of Red Wine Consumption on Serum Oxidation and Adiponectin Levels in Overweight and Healthy Individuals. Pol. J. Food Nutr. Sci..

[79] Xiang L., Xiao L., Wang Y., Li H., Huang Z., He X. (2014). Health benefits of wine: Don’t expect resveratrol too much. Food Chem..

[80] Bresciani L., Calani L., Bocchi L., Delucchi F., Savi M., Ray S., Del Rio D. (2014). Bioaccumulation of resveratrol metabolites in myocardial tissue is dose-time dependent and related to cardiac hemodynamics in diabetic rats. Nutr. Metab. Cardiovasc. Dis..

[81] Chiva-Blanch G., Urpi-Sarda M., Ros E., Valderas-Martinez P., Casas R., Arranz S., Guillén M., Lamuela-Raventós R.M., Llorach R., Andres-Lacueva C. (2013). Effects of red wine polyphenols and alcohol on glucose metabolism and the lipid profile: A randomized clinical trial. Clin. Nutr..

[82] Blomster J.I., Zoungas S., Chalmers J., Li Q., Chow C.K., Woodward M., Neal B. (2014). The Relationship between Alcohol Consumption and Vascular Complications and Mortality in Individuals with Type 2 Diabetes. Diabetes Care.

[83] Chiva-Blanch G., Urpi-Sarda M., Ros E., Arranz S., Valderas-Martinez P., Casas R., Estruch R. (2012). Dealcoholized Red Wine Decreases Systolic and Diastolic Blood Pressure and Increases Plasma Nitric Oxide. Circ. Res..

[84] Bulut D., Jelich U., Dacanay-Schwarz R., Mügge A. (2013). Red Wine Ingestion Prevents Microparticle Formation after a Single High-Fat Meal-A Crossover Study in Healthy Humans. J. Cardiovasc. Pharmacol..

[85] Toth A., Papp J., Rabai M., Kenyeres P., Marton Z., Kesmarky G., Toth K. (2014). The role of hemorheological factors in cardiovascular medicine. Clin. Hemorheol. Microcirc..

[86] Dillenburg D.R., Mostarda C., Moraes-Silva I.C., Ferreira D., Bós D.D.S.G., Duarte A.A.M., Rigatto K. (2013). Resveratrol and grape juice differentially ameliorate cardiovascular autonomic modulation in L-NAME-treated rats. Auton. Neurosci.-Basic Clin..

[87] Levantesi G., Marfisi R., Mozaffarian D., Franzosi M.G., Maggioni A., Nicolosi G.L., Marchioli R. (2013). Wine consumption and risk of cardiovascular events after myocardial infarction: Results from the GISSI-Prevenzione trial. Int. J. Cardiol..

[88] Covas M.I., Gambert P., Fitó M., de la Torre R. (2010). Wine and oxidative stress: Up-to-date evidence of the effects of moderate wine consumption on oxidative damage in humans. Atherosclerosis.

